# Comment to: Socioeconomic inequities in prostate cancer care: private *versus* public treatment settings pose a significant impact on overall survival

**DOI:** 10.31744/einstein_journal/2026CE2346

**Published:** 2026-04-24

**Authors:** Rafael Gomes de Melo D’Elia, Angélica Saiuri de Aurelio Penteado

**Affiliations:** 1 Universidade de São Paulo Hospital das Clínicas Faculdade de Medicina São Paulo SP Brazil Hospital das Clínicas, Faculdade de Medicina, Universidade de São Paulo, São Paulo, SP, Brazil.

Dear Editor,

The article "Socioeconomic inequities in prostate cancer care: private *versus* public treatment settings pose a significant impact on overall survival"^(1)^ addresses an urgent question by comparing outcomes *among* men with metastatic prostate cancer treated within Brazil's public and private healthcare systems at a single tertiary center. The reported 37-month median overall-survival gap (115 *versus* 78 months), along with the finding that patients treated in the public system received fewer treatment lines, is striking and warrants urgent scrutiny.

The unicentric design – using the same staff and protocols with medication availability differing by payer – helps reduce institutional heterogeneity; however, payer status remains a complex proxy for socioeconomic disadvantage. International syntheses demonstrate that insurance/payer type is independently associated with cancer survival across contexts, implying that system-level coverage differences can predict mortality after adjustment for measured covariates.^(2)^ Regional evidence likewise links socioeconomic deprivation to a later stage at presentation and poorer cancer outcomes, supporting the authors’ observation that patients treated in the public system more often present with advanced disease.^(3)^

Access to sequential systemic therapies is a plausible mediator: real-world cohorts document that a substantial proportion of patients with metastatic castration-resistant prostate cancer receive few or no life-prolonging agents, and receipt of additional active lines is associated with markedly longer survival. These patterns underscore how restricted drug availability and delayed incorporation into public formularies may translate into life-years lost.^(4)^ Trial participation, which often affords earlier access to novel agents, is unequally distributed and likely amplifies these gaps; the underrepresentation of minority and socioeconomically disadvantaged groups in prostate cancer trials is well documented.^(5)^

Despite adjustment for some clinical factors, key sources of residual confounding remain. The number of treatment lines is an imperfect proxy for therapeutic value because it does not capture agent identity, sequencing, timing, dose intensity, reasons for discontinuation, or time on the drug – features that ultimately determine clinical benefit. Moreover, referral-selection bias at a tertiary center, along with unmeasured variables (such as metastatic burden, frailty, health literacy, and geographic barriers), limits causal attribution and generalizability beyond this center. Finally, institutional and national processes for drug incorporation entail substantive delays in public systems, strengthening the argument that regulatory and reimbursement dynamics are central to the observed disparity. ^(6)^

Costa et al. have provided an important, policy-relevant signal. To move from description to intervention, we urge the presentation of drug-by-line exposure, time-on-therapy, and reasons for cessation, clinical trial enrollment rates, sensitivity analyses using time-dependent and mediation methods, and replication in multicenter or registry data. Such work would better identify which upstream and downstream levers – diagnostic pathways, formulary timelines, trial access, or social policy measures – should be prioritized to reduce inequitable survival outcomes.

We would also be interested in knowing how Costa et al. propose to use the results of their work and how these inequities might be addressed.

Sincerely,

## Data Availability

The underlying content is contained within the manuscript.
